# The National Pension Fund

**Published:** 1887-04-23

**Authors:** 


### THE NATIONAL PENSION FUND.

Any hospital officials or nurses willing to co-operate in the establishment of this fund, should promptly send their name and address to Mr. Howard J. Collins, Secretary, The Hospitals Association, Norfolk House, Norfolk Street, W.C.

THE interest in this fund steadily grows from week to week. As we have already stated, there are upwards of fifteen thousand nurses alone who might join such a fund, and to whom it could not fail to be of the greatest benefit. If the officials are added, then there must be at least twenty thousand persons who ought to join, and who would greatly benefit by taking this step. Names are being sent in day by day; and it may be interesting to state that eighty names have so far been registered, including those of three hospital secretaries and five matrons. The numbers have been as follows in the weeks ending—26th March, 15 names registered; 2nd April, 33 names; 9th April, 14 names; 16th April, 18 names. It ought to be explained, that in the week ending April 2nd, five lady superintendents sent the names of their staff, which in the case of the Hampstead Infirmary included ten nurses and eight probationers. Will not the readers of THE HOSPITAL take this matter up seriously, and not only register their own names, but use their influence to make the scheme known, and to secure adherents? Many suggestions have been made as to the circulation of papers, posters, and so forth, which mean the expenditure of a considerable sum of money on preliminary expenses. We feel this expenditure should be wholly unnecessary, seeing that every nurse, lady superintendent, or official who is interested in the establishment of the Pension Fund can show their interest, and promote its success, by sending copies of THE HOSPITAL containing particulars of the scheme to those institutions and individuals who ought to be interested, and who may be known to them. We propose to continue to publish the opinions and suggestions which may be sent to us for that purpose, and by continued publicity to do our utmost to induce, say from three to five thousand people, to register their names, when a meeting will be called for the purpose of formulating a scheme for the establishment of a National Pension Fund upon a basis wide enough to include all who have any right or desire to become connected with such a provident institution.

